# Integrin β3 in forebrain *Emx1*-expressing cells regulates repetitive self-grooming and sociability in mice

**DOI:** 10.1186/s12868-022-00691-2

**Published:** 2022-03-05

**Authors:** Andrew J. Lopuch, Brian D. Swinehart, Eden L. Widener, Z. Logan Holley, Katherine M. Bland, Christopher J. Handwerk, Cooper A. Brett, Hollyn N. Cook, Anna R. Kalinowski, Hilda V. Rodriguez, M. Irene Song, George S. Vidal

**Affiliations:** Department of Biology, James Madison University, 951 Carrier Drive, Harrisonburg, VA 22807 USA

**Keywords:** Autism spectrum disorder, Integrin, *Itgb3*, Forebrain, Sociability, Grooming, Self-grooming, Integrin beta 3, Integrin β3

## Abstract

**Background:**

Autism spectrum disorder (ASD) is characterized by repetitive behaviors, deficits in communication, and overall impaired social interaction. Of all the integrin subunit mutations, mutations in integrin β3 (*Itgb3*) may be the most closely associated with ASD. Integrin β3 is required for normal structural plasticity of dendrites and synapses specifically in excitatory cortical and hippocampal circuitry. However, the behavioral consequences of *Itgb3* function in the forebrain have not been assessed. We tested the hypothesis that behaviors that are typically abnormal in ASD—such as self-grooming and sociability behaviors—are disrupted with conditional *Itgb3* loss of function in forebrain circuitry in male and female mice.

**Methods:**

We generated male and female conditional knockouts (cKO) and conditional heterozygotes (cHET) of *Itgb3* in excitatory neurons and glia that were derived from *Emx1*-expressing forebrain cells during development. We used several different assays to determine whether male and female cKO and cHET mice have repetitive self-grooming behaviors, anxiety-like behaviors, abnormal locomotion, compulsive-like behaviors, or abnormal social behaviors, when compared to male and female wildtype (WT) mice.

**Results:**

Our findings indicate that only self-grooming and sociability are altered in cKO, but not cHET or WT mice, suggesting that *Itgb3* is specifically required in forebrain *Emx1*-expressing cells for normal repetitive self-grooming and social behaviors. Furthermore, in cKO (but not cHET or WT), we observed an interaction effect for sex and self-grooming environment and an interaction effect for sex and sociability test chamber.

**Limitations:**

While this study demonstrated a role for forebrain *Itgb3* in specific repetitive and social behaviors, it was unable to determine whether forebrain *Itgb3* is required for a preference for social novelty, whether cHET are haploinsufficient with respect to repetitive self-grooming and social behaviors, or the nature of the interaction effect for sex and environment/chamber in affected behaviors of cKO.

**Conclusions:**

Together, these findings strengthen the idea that *Itgb3* has a specific role in shaping forebrain circuitry that is relevant to endophenotypes of autism spectrum disorder.

**Supplementary Information:**

The online version contains supplementary material available at 10.1186/s12868-022-00691-2.

## Background

Several mutations in integrin β3 (*Itgb3*) or loci containing *Itgb3* appear to be associated with autism spectrum disorder [4, 9–11, 27, 32, 37, 39, 40, 43, 45, 51]. Integrins are heterodimeric cell adhesion molecules comprising an alpha and beta subunit that typically bind to the extracellular matrix and regulate cell motility [20]. The integrin subunit that may be most closely associated with autism spectrum disorder is *Itgb3*, and comorbidities of *Itgb3* variants may include echolalia [43] and attention deficit hyperactivity disorder [11]. Altering *Itgb3* function leads to anatomical changes in multiple brain regions [12, 46] as well as anatomical and functional impairments in many neural cell types, including excitatory neurons of the hippocampus [7, 8, 41] and cortex [21, 48], and in the midbrain [11, 28]. Many of these impairments are related to autism spectrum disorder. For example, excitatory cortical neurons with *Itgb3* loss-of-function exhibit lower network synchrony [21] and altered dendritic spine density and dendritic arborization in vivo [48]. Midbrain synapses with *Itgb3* loss-of-function exhibit lowered serotonin transporter activity in vivo [11, 28]. Excitatory hippocampal pyramidal neurons with *Itgb3* loss-of-function show altered AMPA receptor subunit composition [8, 41], NMDA receptor subunit composition and probability of release [7], as well as loss of homeostatic plasticity [8].

Mice with global (i.e., full-body) alterations in *Itgb3* function have deficits in self-grooming, social, and other behaviors [5, 11, 28, 31, 50]. Self-grooming behaviors are driven by excitatory cortical pyramidal neurons [1, 3] and excitatory hippocampal pyramidal neurons [36]. Social behaviors require normal excitatory cortical circuitry [44]. However, the role of *Itgb3* in excitatory forebrain circuitry for all these behaviors is unknown.

We previously demonstrated that *Emx1*-Cre-mediated excision of *Itgb3* reduces integrin β3 expression in the cerebral cortex [48]. *Emx1*-expressing cells include nearly all excitatory pyramidal neurons in cortex and hippocampus [15]. We tested the hypothesis that *Itgb3* is necessary in forebrain *Emx1*-expressing cells for self-grooming, social, and other behaviors by using a conditional knockout strategy. The primary benefit of this strategy was to dissect the function of *Itgb3* across various cell types and brain regions. Another advantage was that *Emx1* is not expressed in platelets, where *Itgb3* is required for platelet aggregation, so we did not experience any platelet-related hemorrhaging and survivability issues that have plagued the global *Itgb3* knockout in the past [5, 17]. Additionally, prior behavioral studies on mice with *Itgb3* loss-of-function have not explicitly included females, even though women with certain *Itgb3* mutations may be at an increased risk of autism spectrum disorder [51]. So, here we tested both male and female mice. We utilized three groups to determine whether *Itgb3* is necessary in forebrain *Emx1*-expressing cells for normal behaviors: wildtype (WT), conditional heterozygotes (cHET), and conditional knockouts (cKO). Conditional knockout of *Itgb3* from one allele (cHET) or both alleles (cKO) was achieved by crossing floxed *Itgb3* mice to the *Emx1-Cre* line, targeting mostly excitatory cortical and hippocampal neurons [15] and decreasing integrin β3 protein levels [48]. Our results show that *Itgb3* is necessary in forebrain *Emx1*-expressing cells for normal self-grooming and sociability behaviors, and that there is an interaction of sex and environment/chamber on cKO in these behaviors.

## Results

To control for the expression of Cre recombinase in some of the mice, we compared WT Cre− to WT Cre+ mice, and no differences were observed between these two groups in any behavioral measures (Additional file 2: Table S5). We also observed that cKO and cHET mice do not hemorrhage or have lower survival than WT, which contrasts with full-body *Itgb3* knockout mice [5, 17]. Furthermore, the overall gross morphology of the brain is unaffected in cKO and cHET mice when compared to WT mice (Additional file 1: Fig. S1, Additional file 2: Table S6). We examined adult male and female cKO, cHET, and WT mice in a series of behavioral tests, most of which have been performed on full-body *Itgb3* knockout and/or heterozygous knockout mice [5, 11, 31, 50].

**Fig. 1:**
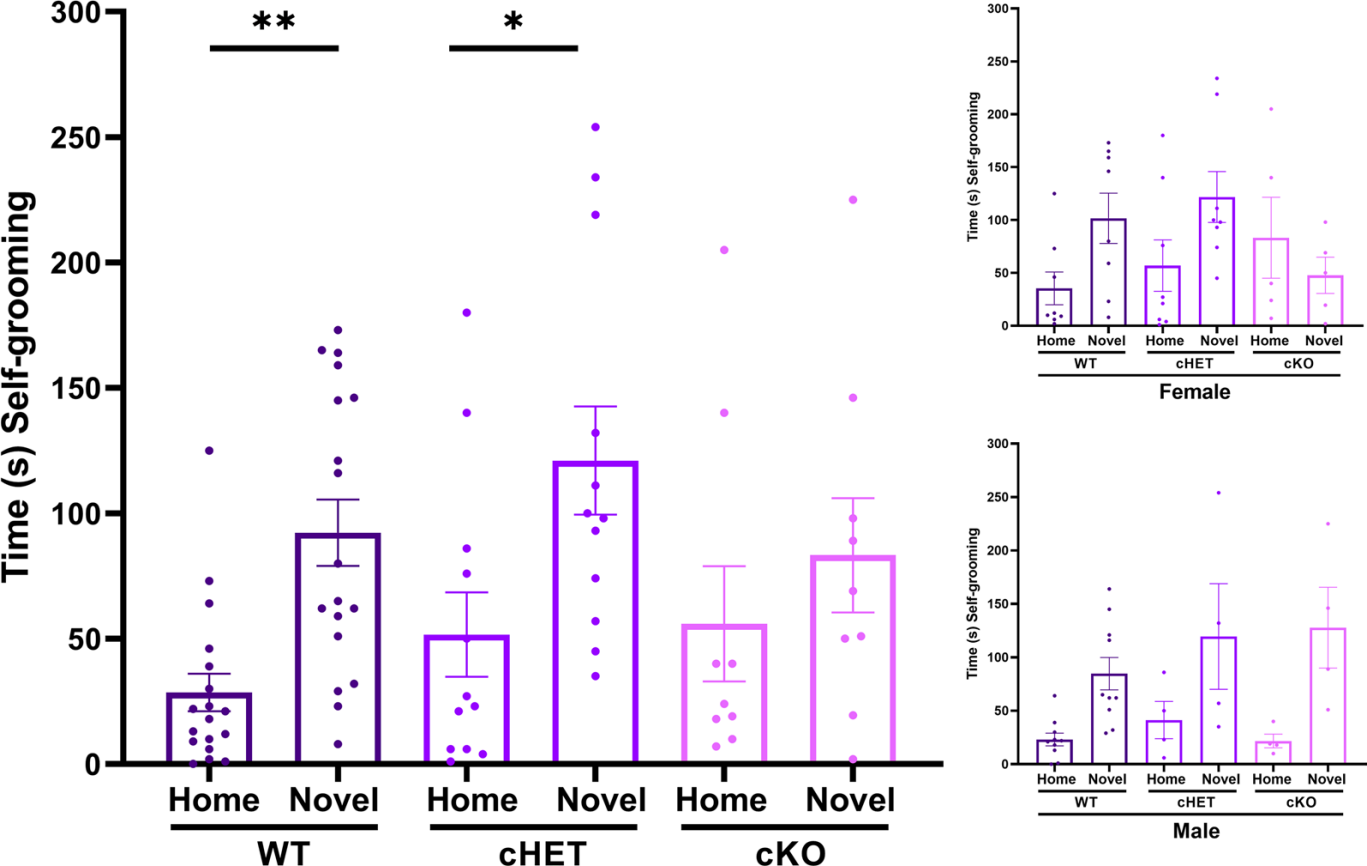
Self-grooming times for WT, cHET and cKO mice in a home cage and novel environment. Self-grooming times are different in novel versus home environments overall (p = 0.0002, two-way ANOVA). WT and cHET mice exhibit differences in grooming behavior in a novel versus home environment (** p = 0.0049, * p = 0.0143, Šidák's multiple comparisons) while cKO mice do not (p = 0.6896, Šidák's multiple comparisons). Insets: Female (top) and male (bottom) grooming times in WT, cHET and cKO groups. See Table 1 and Additional file 2: Table S1 for means ± SEM

Full-body *Itgb3* knockout mice are known to self-groom more than WT in a novel environment, but not in their home environment [5]. We therefore designed our self-grooming tests to directly compare the difference in self-grooming times between novel and home environments across genotypes. When all experimental mice were taken as a whole, we observed a significant effect of the environment (home versus novel) on self-grooming times (p < 0.0002, Fig. 1, Table 1). As expected, longer self-grooming times were noted for mice in the novel environment overall (Fig. 1, Table 1, Additional file 2: Table S1). Within-sex ANOVA revealed that there was a significant effect of environment on self-grooming times among male mice (p < 0.0001) but not among female mice (p > 0.12), when taken as a whole (Additional file 2: Table S1). Within-genotype ANOVA revealed that there was a significant effect of environment on self-grooming times in WT (p = 0.0003) and cHET mice (p = 0.029), but not cKO mice (p > 0.24; Additional file 2: Table S1). Indeed, a post-hoc test showed that self-grooming was higher in novel environments when compared to home environments in WT (p = 0.0049) and cHET (p = 0.014) mice, but not in cKO mice (p > 0.68; Fig. 1, Table 1). Among cKO mice, there was a significant interaction between sex and environment in self-grooming times (p = 0.029; Additional file 2: Table S1). When all experimental mice were taken as a whole, no significant interactions were observed among all three factors of genotype, sex, and environment (p > 0.08; Additional file 2: Table S1). Overall, our results show that WT and cHET mice self-groomed more in novel versus home environments while cKO mice did not (Fig. 1, Table 1), and that there was a significant interaction factor between sex and environment only in cKO (Additional file 2: Table S1).

**Table 1:** Self-grooming

	Factor	DFn	DFd	F	p	* Šidák's multiple comparisons	p
Two-way ANOVA	Genotype	2	72	1.426	0.2469	WT, Home vs WT, Novel	**0.0049**
	Environment	1	72	**15.11**	0.0002*	cHET, Home vs cHET, Novel	**0.0143**
	Interaction	2	72	0.7727	0.4656	cKO, Home vs cKO, Novel	0.6896

	Factor	DFn	DFd	F	p	* Šidák's multiple comparisons	p
Two-way ANOVA	Environment	1	74	**19.36**	< 0.0001*	Female, Home vs Female, Novel	**0.0475**
	Sex	1	74	0.7243	0.3975	Male, Home vs Male, Novel	**0.0005**
	Interaction	1	74	1.613	0.2081		

	Factor	DFn	DFd	F	p	* Šidák's multiple comparisons	p
Two-way ANOVA	Genotype	2	72	0.8625	0.4264		
	Sex	1	72	0.08923	0.766		
	Interaction	2	72	0.1984	0.8205		

	Genotype: WT			Genotype: cHET			Genotype: cKO		
	Environment	n	Mean ± SEM	Environment	n	Mean ± SEM	Environment	n	Mean ± SEM
N, mean, SEM	Home	18	28.56 ± 7.50	Home	12	51.67 ± 16.84	Home	9	55.89 ± 23.01
	Novel	18	92.22 ± 13.23	Novel	12	121.00 ± 21.59	Novel	9	83.27 ± 22.76

Two-way ANOVA analyses of genotype (WT, cHET, cKO), environment (home, novel), and/or sex (female, male) factors in self-grooming behaviors. DFn = Degrees of freedom numerator (between-subject degrees of freedom—1); DFd = Degrees of freedom denominator (within-subject degrees of freedom—between-subject degrees of freedom). See Additional file 2: Table S1 for within-group ANOVA comparisons and within-group N, Mean ± SEM

**Fig. 2:**
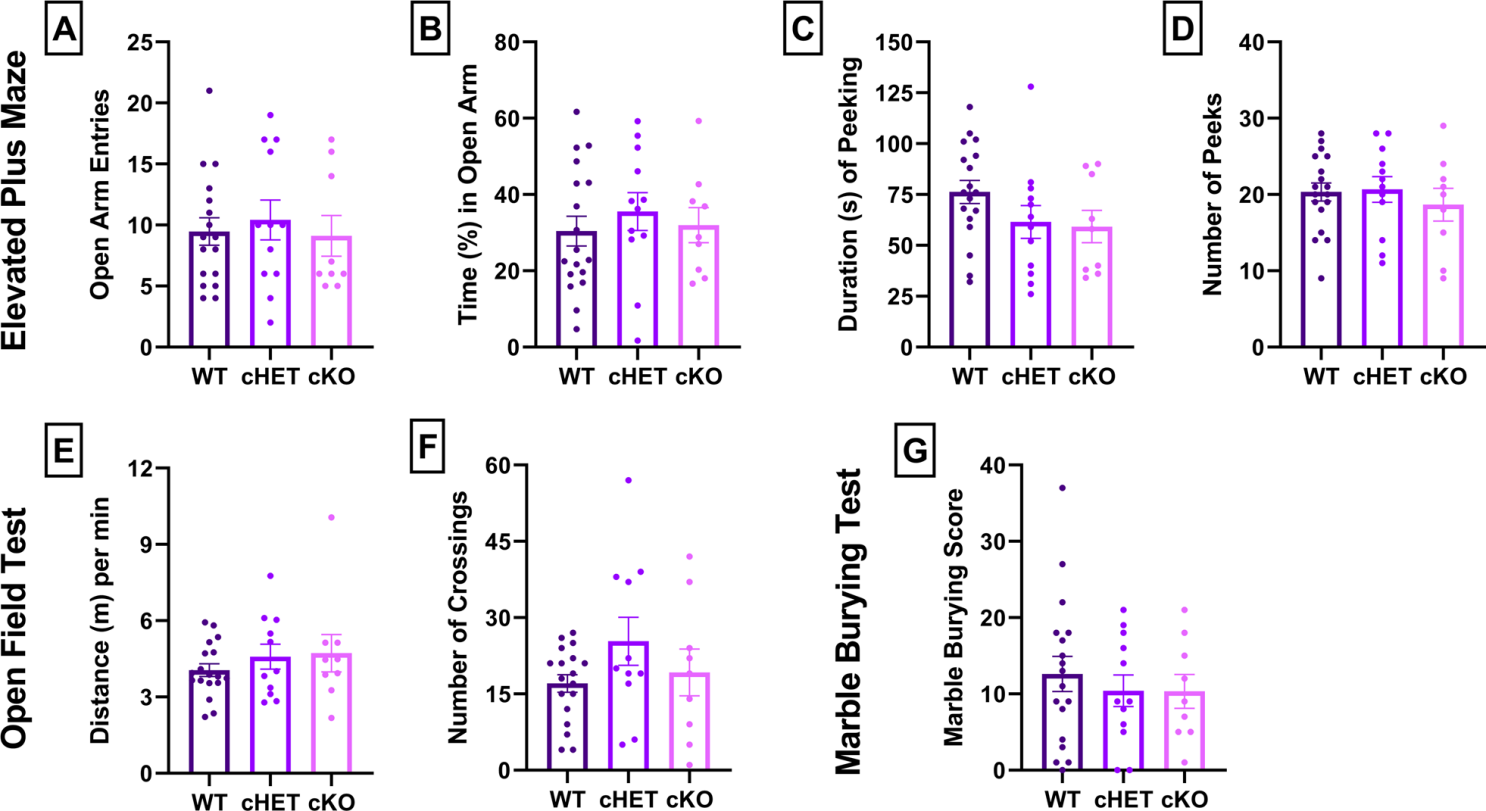
Elevated plus maze (EPM), open field test (OFT) and marble burying test (MBT) results among WT, cHET and cKO mice. All p > 0.13 (two-way ANOVA); see Table 2 for means ± SEM

Increased self-grooming in a novel versus home environment could arise from changes in anxiety, locomotion, or compulsive behaviors. The elevated plus maze (EPM) was used to test general anxiety levels, the open field test (OFT) was used to test general anxiety as well as locomotion, and the marble burying test (MBT) was used to measure compulsive behaviors. There were no significant effects of genotype, sex, or their interactions in any EPM, OFT, or MBT measure (p > 0.13; Fig. 2, Table 2, Additional file 2: Table S2).

**Table 2:** EPM, OFT, and MBT

	Experiment: Fig. 2A. EPM open arm entries					Experiment: Fig. 2B. EPM percent time in open arm				
	Factor	DFn	DFd	F	p	Factor	DFn	DFd	F	p
Two-way ANOVA	Genotype	2	33	0.09343	0.9110	Genotype	2	33	0.1993	0.8203
	Sex	1	33	1.178	0.2857	Sex	1	33	0.8636	0.3595
	Interaction	2	33	0.07359	0.9292	Interaction	2	33	0.006488	0.9935

	Experiment: Fig. 2C. EPM duration of peeking behaviors (s)					Experiment: Fig. 2D. EPM number of peeking behaviors				
	Factor	DFn	DFd	F	p	Factor	DFn	DFd	F	p
Two-way ANOVA	Genotype	2	33	1.384	0.2647	Genotype	2	33	0.3888	0.6810
	Sex	1	33	1.627	0.2110	Sex	1	33	0.03200	0.8591
	Interaction	2	33	0.2435	0.7853	Interaction	2	33	0.09161	0.9127

	Experiment: Fig. 2E. OFT distance traveled (m) per minute					Experiment: Fig. 2F. OFT number of crossings				
	Factor	DFn	DFd	F	p	Factor	DFn	DFd	F	p
Two-way ANOVA	Genotype	2	33	0.4342	0.6516	Genotype	2	33	1.319	0.2815
	Sex	1	33	2.333	0.1365	Sex	1	33	0.1444	0.7064
	Interaction	2	33	0.2352	0.7918	Interaction	2	33	1.172	0.3227

	Experiment: Fig. 2G. Marble burying score									
	Factor	DFn	DFd	F	p					
Two-way ANOVA	Genotype	2	33	0.1674	0.8466					
	Sex	1	33	1.688	0.2029					
	Interaction	2	33	0.003392	0.9966					

	Genotype: WT			Genotype: cHET			Genotype: cKO			
	Experiment	n	Mean ± SEM	Experiment	n	Mean ± SEM	Experiment	n	Mean ± SEM	
N, mean, SEM	Fig. 2A. EPM Open Arm Entries	18	9.47 ± 1.13	Fig. 2A. EPM Open Arm Entries	12	10.42 ± 1.63	Fig. 2A. EPM Open Arm Entries	9	9.11 ± 1.67	
	Fig. 2B. EPM Percent Time in Open Arms	18	30.41 ± 3.88	Fig. 2B. EPM Percent Time in Open Arms	12	35.55 ± 4.95	Fig. 2B. EPM Percent Time in Open Arms	9	31.98 ± 4.61	
	Fig. 2C. EPM Duration of Peeking Behaviors (s)	18	76.28 ± 5.67	Fig. 2C. EPM Duration of Peeking Behaviors (s)	12	61.50 ± 8.11	Fig. 2C. EPM Duration of Peeking Behaviors (s)	9	59.22 ± 7.91	
	Fig. 2D. EPM Number of Peeking Behaviors	18	20.33 ± 1.19	Fig. 2D. EPM Number of Peeking Behaviors	12	20.67 ± 1.68	Fig. 2D. EPM Number of Peeking Behaviors	9	18.67 ± 2.16	
	Fig. 2E. OFT Distance Traveled (m) per Minute	18	4.05 ± 0.25	Fig. 2E. OFT Distance Traveled (m) per Minute	12	4.59 ± 0.50	Fig. 2E. OFT Distance Traveled (m) per Minute	9	4.72 ± 0.74	
	Fig. 2F. OFT Number of Crossings	18	17.06 ± 1.72	Fig. 2F. OFT Number of Crossings	12	25.36 ± 4.73	Fig. 2F. OFT Number of Crossings	9	19.22 ± 4.61	
	Fig. 2G. Marble Burying Score	18	12.61 ± 2.30	Fig. 2G. Marble Burying Score	12	10.42 ± 2.07	Fig. 2G. Marble Burying Score	9	10.33 ± 2.22	

Two-way ANOVA analyses of genotype (WT, cHET, cKO) and sex (female, male) factors in EPM, OFT, and MBT behaviors. See Additional file 2: Table S2 for within-group ANOVA comparisons and within-group N, Mean ± SEM

**Fig. 3:**
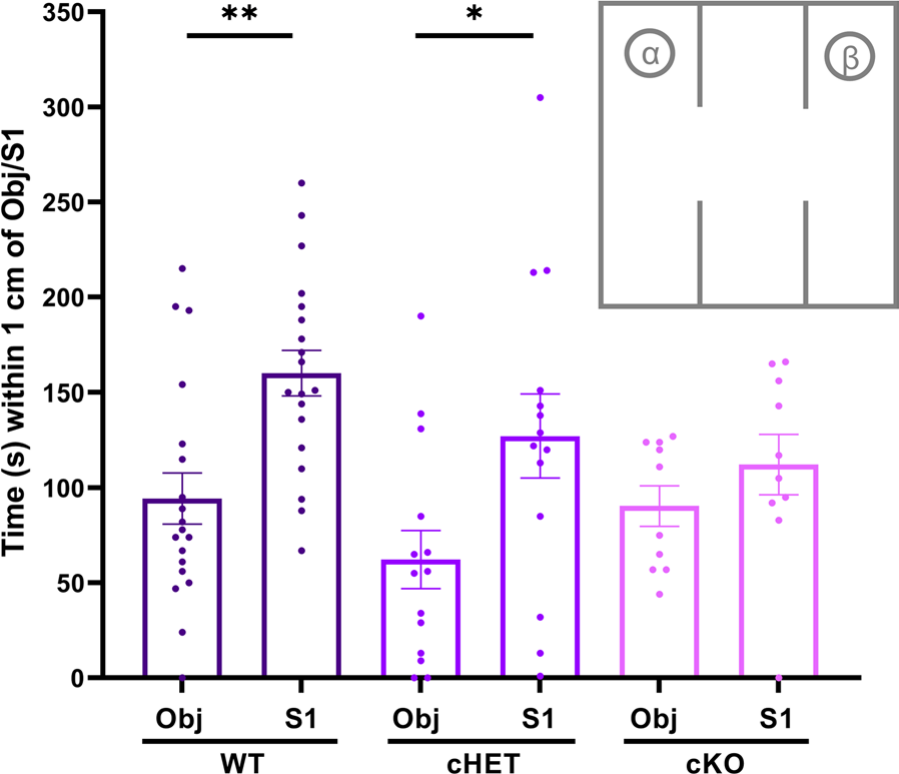
Sociability test: time spent within 1 cm of a novel object (Obj) or a stranger mouse (S1). Times spent near Obj vs S1 are different overall (p = 0.0002, two-way ANOVA). WT and cHET mice exhibit differences in time spent with Obj vs S1 (i.e., “are sociable”; **p = 0.0026, *p = 0.0132, Šidák's multiple comparisons) while cKO mice do not (p = 0.7917, Šidák's multiple comparisons). See Table 3 for means ± SEM. *Inset*: Schematic (not to scale) of the experimental setup for sociability experiments. An object (Obj) was placed in one of the circles marked “α” or “β”, and a stranger mouse (S1) was kept inside the other circle. Experimental mice had the freedom to move around all three chambers

Full-body *Itgb3* knockout mice have intact sociability but decreased preference for social novelty when compared to WT [5]. In other words, full-body *Itgb3* knockout mice generally prefer exploring a new stranger mouse (“Stranger 1”, “S1”) over a new object (“Object”, “Obj”), a behavior called “sociability”. These same knockout mice, however, do not prefer exploring a new stranger mouse (“Stranger 2”, “S2”) over the stranger (S1) they just interacted with, a behavior called “preference for social novelty”. In a similar three-chambered apparatus, we measured the amount of time mice spent in a side chamber within 1 cm of a novel object (Obj) or in the opposite side chamber within a WT stranger mouse of the same sex (S1). When all experimental mice were taken as a whole, we observed that what was in the chamber (Obj versus S1) had a significant effect on mouse behavior (p < 0.0002; Fig. 3, Table 3). As expected, mice overall spent more time within 1 cm of Stranger 1 than within 1 cm of the Object (Fig. 3, Table 3, Additional file 2: Table S3). Within-sex ANOVA revealed that there was a significant effect of Obj versus S1 among male mice (p < 0.0001) but not among female mice (p > 0.13), when taken as a whole (Additional file 2: Table S3), and in WT, there was an effect of sex on total interaction times (p = 0.035). Within-genotype ANOVA revealed that there was a significant effect of Obj/S1 among WT (p = 0.0008) and cHET mice (p = 0.025), but not among cKO mice (p > 0.23; Additional file 2: Table S3). A post-hoc test showed that mice spent more time within 1 cm of Stranger 1 than within 1 cm of the Object among WT (p = 0.0026) and cHET mice (p = 0.013), but not among cKO mice (p > 0.79, Fig. 3, Table 3). Finally, among cKO, there was a significant interaction between sex and time spent with Obj versus S1 (p = 0.048, Additional file 2: Table S3). When all experimental mice were taken as a whole, no significant interactions were observed among all three factors of genotype, sex, and chamber (p > 0.57; Additional file 2: Table S3). Overall, our results show that WT and cHET mice spent more time within 1 cm of Stranger 1 than within 1 cm of the Object, while cKO mice did not (Fig. 3, Table 3), and that there was a significant interaction factor between sex and chamber only in cKO (Additional file 2: Table S3).

**Table 3:** Sociability

	Factor	DFn	DFd	F	p	*Šidák's multiple comparisons	p
Two-way ANOVA	Genotype	2	80	2.811	0.0661	WT, Object vs WT, Stranger 1	**0.0026**
	Chamber	1	80	**15.12**	0.0002*	cHET, Object vs cHET, Stranger 1	**0.0132**
	Interaction	2	80	1.061	0.3508	cKO, Object vs cKO, Stranger 1	0.7917

	Factor	DFn	DFd	F	p	*Šidák's multiple comparisons	p
Two-way ANOVA	Chamber	1	82	**18.23**	< 0.0001*	Female, Object vs Female, Stranger 1	0.06
	Sex	1	82	1.43	0.2353	Male, Object vs Male, Stranger 1	**0.0005**
	Interaction	1	82	1.252	0.2664		

	Factor	DFn	DFd	F	p	*Šidák's multiple comparisons	p
Two-way ANOVA	Genotype	2	80	2.905	0.0605		
	Sex	1	80	0.9929	0.322		
	Interaction	2	80	1.033	0.3605		

	Genotype: WT			Genotype: cHET			Genotype: cKO		
	Time (s) near…	n	Mean ± SEM	Time (s) near…	n	Mean ± SEM	Time (s) near…	n	Mean ± SEM
N, mean, SEM	Object	19	94.32 ± 13.45	Object	14	62.29 ± 15.28	Object	10	90.40 ± 10.63
	Stranger 1	19	160.0 ± 11.97	Stranger 1	14	127.1 ± 21.93	Stranger 1	10	112.2 ± 15.91

Two-way ANOVA analyses of genotype (WT, cHET, cKO), “chamber” (Object or Stranger 1), and/or sex (female, male) factors in sociability behaviors. See Additional file 2: Table S3 for within-group ANOVA comparisons and within-group N, Mean ± SEM

Surprisingly, there was no overall preference for social novelty (p > 0.05, Table 4), even among male WT mice (Table 4; Additional file 2: Table S4). We also attempted to analyze sociability and preference for social novelty in an alternative way, by measuring the time spent in each side chamber, rather than time spent near (< 1 cm) the object or stranger mice. However, this analysis did not demonstrate a significant effect of chamber on mouse behavior in the sociability test (p > 0.08, Additional file 2: Table S7), as was shown when analyzing by time spent near the object or stranger mice (p < 0.0002, Table 3). Instead, we found an interaction between sex and chamber preference (p = 0.0085, Additional file 2: Table S7), and a post-hoc test showed that males showed an overall chamber preference (p = 0.024), but females did not (p > 0.9, Additional file 2: Table S7). In the preference for social novelty test, we also found an interaction between sex and chamber preference (p = 0.046, Additional file 2: Table S8), and females showed an overall chamber preference (p = 0.037) whereas males did not (p > 0.7, Additional file 2: Table S7). There was also a significant effect of chamber side (p = 0.045) and an interaction between genotype and chamber preference (p = 0.021, Additional file 2: Table S8). A post-hoc test showed that cKO (p = 0.020), but not cHET (p > 0.5) or WT (p > 0.7), had a chamber preference, with cKO spending more time in the chamber with S2 rather than the chamber with S1. There were no differences between the number of trips taken into the two side chambers in any condition (Additional file 2: Tables S7, S8). Furthermore, mice for all groups spent significantly less time in the middle chamber than in the side chambers (Additional file 2: Tables S7, S8), demonstrating that mice had an overall preference for the side chambers, which contained novel objects and stranger mice. As expected with the layout of the three-chambered box, mice took trips to the middle chamber more than the side chambers (Additional file 2: Tables S7, S8), which corroborated our observation that all mice were freely exploring during the test period, rather than engaging in non-exploratory behaviors (e.g., self-grooming).

**Table 4:** Preference for social novelty

	Factor	DFn	DFd	F	p
Two-way ANOVA	Genotype	2	78	1.358	0.2631
	Chamber	1	78	0.7936	0.3758
	Interaction	2	78	1.546	0.2195

	Factor	DFn	DFd	F	p
Two-way ANOVA	Chamber	1	80	0.5556	0.4582
	Sex	1	80	0.1239	0.7257
	Interaction	1	80	3.723	0.0572

	Factor	DFn	DFd	F	p
Two-way ANOVA	Genotype	2	78	1.278	0.2845
	Sex	1	78	0.04568	0.8313
	Interaction	2	78	0.1424	0.8675

	Genotype: WT			Genotype: cHET			Genotype: cKO		
	Time (s) near…	n	Mean ± SEM	Time (s) near…	n	Mean ± SEM	Time (s) near…	n	Mean ± SEM
N, mean, SEM	Stranger 1	19	101.0 ± 12.07	Stranger 1	13	91.46 ± 23.19	Stranger 1	10	68.46 ± 14.98
	Stranger 2	19	113.0 ± 14.17	Stranger 2	13	71.85 ± 12.83	Stranger 2	10	113.5 ± 23.79

Two-way ANOVA analyses of genotype (WT, cHET, cKO), “chamber” (Object or Stranger 1), and/or sex (female, male) factors in preference for social novelty behaviors. See Additional file 2: Table S4 for within-group ANOVA comparisons and within-group N, Mean ± SEM

We also collected data on sociability and preference for social novelty with opposite-sex mice (Additional file 2: Tables S7, S8). Interestingly, sociability and preference for social novelty results were similar to those of same-sex experiments (Tables 3, 4): In the sociability test, what was in the chamber (Obj versus S1) had a significant effect on mouse behavior (p < 0.0001), with mice spending more time with the opposite-sex S1 (Additional file 2: Table S9). Post-hoc tests showed that mice spent more time within 1 cm of the opposite-sex S1 than within 1 cm of the Object among both females (p = 0.021) and males (p = 0.0003), among WT (p = 0.0052) and cHET mice (p = 0.015), but not among cKO mice (p > 0.28, Additional file 2: Table S9). As with same-sex experiments (Table 4), there was no overall preference for social novelty in the opposite-sex paradigm (p > 0.08, Additional file 2: Table S10). Table 5 shows where our data are presented in this article.

**Table 5:** Location of presented data in the article, organized by experiment

Experiment	Graphical representation	Overall N, mean, SEM. If applicable: two-way ANOVAs, Šidák's multiple comparisons	If applicable: Within-category ANOVAs, N, mean, SEM, three-way ANOVA
Grooming	Fig. 1	Table 1	Additional file 2: Table S1
EPM/OFT/MBT	Fig. 2	Table 2	Additional file 2: Table S2
Sociability	Fig. 3	Table 3	Additional file 2: Table S3
Pref Soc Novelty		Table 4	Additional file 2: Table S4
Cre + vs Cre−		Additional file 2: Table S5	
Brain morphology	Additional file 1: Fig. S1	Additional file 2: Table S6	
Sociability (by chamber)		Additional file 2: Table S7	
Preference for social novelty (by chamber)		Additional file 2: Table S8	
Sociability (opposite sex)		Additional file 2: Table S9	Additional file 2: Table S9
Preference for social novelty (opposite sex)		Additional file 2: Table S10	Additional file 2: Table S10

## Discussion

We showed that cKO mice, but not cHET mice, had two clear behavioral deficits in this study. First, we demonstrated that WT and cHET mice self-groom more in a novel versus home environment while cKO mice do not (Fig. 1, Table 1, Additional file 2: Table S1). Second, we showed that sociability was intact in WT and cHET but not cKO mice, regardless of the sex of the stranger mice (Fig. 3, Table 3, Additional file 2: Table S3, Additional file 2: Table S9).

This study had the distinct advantage of preventing what Carter et al. [5] called the “peripheral phenotype” of complete *Itgb3* loss-of-function: bleeding, hemorrhage, and low survival [17]. Carter et al. [5] showed that full-body *Itgb3* knockout mice self-groomed more in a novel environment when compared to wildtypes and heterozygotes. It is well known that exposure to novel environments and other stress inducers increase self-grooming tendencies [23]. Like Carter et al. [5], we also detected an *Itgb3*-dependent change in this behavior, albeit not in the exact same measure: We observed that, while self-grooming times increased as expected in novel versus home environments for WT mice, they did not increase for cKO mice. Self-grooming behaviors, especially those involving novel and stress-induced situations, are modulated by a wide variety of brain regions, including forebrain circuitry [24]. The amygdala is home to a sparse population of *Emx1*-expressing neurons [15], and an optogenetic study has shown that activating *vGlut2*-positive neurons in the amygdala directly promotes self-grooming behavior [19], but its role in stress-induced self-grooming was not directly assessed. A recent optogenetic study directly linked stress-induced self-grooming behavior to a specific, disynaptic circuit that involves glutamatergic neurons of the hippocampal formation (ventral subiculum) that project to GABAergic neurons of the ventral lateral septum, which then project to the lateral hypothalamus tuberal nucleus [36]. The first neurons in this circuit (glutamatergic neurons in the ventral subiculum) could have been modified directly by *Itgb3* loss of function in the cKO, because *Emx1* expression is very high in the subiculum and in glutamatergic pyramidal neurons, while *Emx1* expression is practically nonexistent in GABAergic neurons and in the hypothalamus [15]. Moreover, *Itgb3* loss-of-function is known to lead to significant anatomical [48] and functional [7, 8, 21, 41] deficits in glutamatergic pyramidal neurons. Further identifying the circuitry that is most affected by *Itgb3* loss of function in *Emx1*-expressing cells (*i.e.*, cKO), could reveal an extremely specific role for *Itgb3* function in repetitive grooming behaviors.

In the EPM and OFT, our results match those of prior studies on full-body *Itgb3* knockout mice [5, 31], with two exceptions: McGeachie et al. [31] noted an increase for full-body *Itgb3* knockout mice in middle crossings of the OFT and in open EPM arm entries. The authors surmised that the difference between their results and those of Carter et al. [5] could have been to genetic background. We agree with this assessment, as both the genetic background of our mice (C57BL6/J) was that of Carter et al. [5], and the results of the EPM and OFT were most similar to that study. In the sociability test, our results contrast with Carter et al. [5] in which *Itgb3* did not seem to be necessary for normal sociability. Taking these results at face value, one simplistic explanation is that the full-body *Itgb3* knockout had additional dysfunctions in non-*Emx1*-expressing circuitry that had the effect of promoting sociability, but what these precise circuits could be is unknown.

Sociability, which was deficient in cKO mice, is a behavioral trait that involves excitatory prefrontal cortical circuitry and is commonly disrupted in mouse models of autism spectrum disorder. For example, an aberrant increase in excitatory-inhibitory balance in the CNTNAP2 mouse was corrected by reducing the firing rate of glutamatergic pyramidal neurons in the medial prefrontal cortex, rescuing normal social behavior [44]. Similarly, activity of glutamatergic pyramidal neurons of the anterior cingulate cortex is required for normal social behavior, and these neurons are primarily affected in the *Shank3* knockout model of autism spectrum disorder [16]. Because *Emx1* expression is concentrated in glutamatergic pyramidal neurons of the cortex and hippocampus, it is likely that the deficit in sociability we observed in cKO mice involves a similar glutamatergic cortical circuit. Multiple optogenetic studies have now begun to dissect precise, monosynaptic cortical circuitry involved in sociability. For example, activity of deep-layer prefrontal cortical pyramidal neurons that project to the posterior paraventricular thalamus [53], basolateral amygdala [25], and lateral habenula [2] all directly modulate sociability. In all of these studies, modulating these prefrontal cortical circuits led to immediate changes in sociability, without adversely affecting locomotion, exploration, or anxiety behaviors. In our study, cKO mice had defective sociability without changes in locomotion, exploration, or anxiety behaviors. Because it is now known that *Itgb3* loss-of-function in cortical pyramidal neurons leads to anatomical [48] and functional [21] deficits, we would predict that *Itgb3* is involved in shaping at least one of these specific prefrontal circuits that modulate sociability.

We detected a modest interaction effect between sex and environment or chamber within cKO (but not cHET or WT) in self-grooming and sociability behaviors, respectively (Additional file 2: Tables S1, S3). Informally comparing the means of cKO performance in the self-grooming and sociability tests would suggest the tantalizing possibility that female cKO but not male cKO display aberrant behavior. We caution readers, however, that our experiments were only designed to test the hypothesis of the existence of an interaction effect for sex and environment in self-grooming and for sex and sociability chamber in cKO behaviors (a two-way interaction), rather than the precise nature of that relationship. In other words, we can conclude that there is an effect of sex on self-grooming and sociability behaviors in cKO, but we cannot conclude about the nature of that effect. Among the several studies demonstrating a possible relationship between human *Itgb3* mutations and ASD, six explicitly included both male and female data [4, 11, 27, 32, 45, 51] and, except for Ma et al*.* [27], found that sex was an important factor in their analyses. These results generally match the well-known sex bias in ASD. It is known that sex differences in steroid expression and that inflammatory molecules regulate brain development (reviewed by [29]). It has been proposed that masculinization of the brain may cause it to become more vulnerable to inflammation, leading to ASD [29]. Integrins are involved in some inflammatory and injury-related pathways in the brain [22]. Specifically, integrin β3 expression and activation in the central nervous system can be modulated by molecules in these pathways. For example, the inflammatory cytokine tumor necrosis factor α (TNFα)—which is required for activity-dependent synaptic scaling [47]—increases integrin β3 levels of hippocampal pyramidal neurons in vitro [8]. Fibrinogen, a molecule released during injury, is required for regulating glycine receptor dynamics at inhibitory synapses of spinal cord neurons in vitro [6]. How integrin β3 and TNFα/fibrinogen interactions may be modulated by sex differences in brain development, however, is unknown.

### Limitations

Our study is limited in that it could not (1) rule out small-scale changes in brain region volumes in cHET or cKO mice or the precise location of decreased integrin β3 expression, (2) test whether cHET mice were haploinsufficient for *Itgb3*, (3) test for preference for social novelty, (4) describe the nature of opposite-sex versus same-sex sociability and preference for social novelty, or the nature of the interaction between sex and *Itgb3* genotype, or (5) eliminate the possibility that cHET and even cKO were deficient in other, untested behaviors.

Although we can conclude that the overall gross morphology of the brain is unaffected in cKO and cHET mice when compared to WT mice (Additional file 1: Fig. S1, Additional file 2: Table S6), we cannot rule out that the total volume of specific brain regions is unaffected. For example, prior work showed volume changes of various brain regions in the full-body *Itgb3* knockout that ranged from a 12.8% reduction to 7.8% increase [12]. Additionally, this study was not designed to determine where integrin β3 protein is decreased in experimental mice. Although the targeting of the *Emx1*-Cre line has been characterized [15], and although we know that integrin β3 protein expression is decreased in cKO cerebral cortex [48], the expression pattern of integrin β3 in the forebrain (and its expression pattern after conditional knockout) is presently uncharacterized.

*Itgb3* haploinsufficiency is known to affect cortical network activity in vitro [21]. In this study, we tested cHET mice, in which one copy of *Itgb3* is deleted from *Emx1*-expressing cells of the forebrain, and found that they do not share any of the deficits seen in cKO mice. However, we caution readers that we did not directly test for haploinsufficiency of *Itgb3*. This is because our experimental design treated cHET as a separate experimental group. It is tantalizing that many of the overall reported means and even statistics (e.g., p-values) appear to show cHET data lying “in between” that of cKO and WT, but it would be fallacious to conclude anything about the haploinsufficiency of cHET mice for this reason [38].

Preference for social novelty was disrupted in a previous study on full knockouts of *Itgb3* [5]. It was surprising to see that none of the three groups tested displayed a preference for social novelty, since this is a behavior seen in most WT mice [5, 34]. Previous experimental designs measured the time spent within 1 cm of each condition [5] or the time spent in each chamber [34]. Our study measured both. However, in previous studies where preference for social novelty was detected, chambers were 40–45 cm smaller in length and 20–25 cm smaller in width [5, 34]. Between these two experimental setups that showed preference for social novelty, the largest effect was seen in the study using the smallest chamber Moy et al., [34]. Furthermore, our chambers were composed of opaque acrylic, whereas prior studies used transparent acrylic [34] or (presumably clear) polycarbonate [5]. In the sociability and preference for social novelty tests, we also analyzed the time spent in the side chambers containing Obj/S1 or S1/S2, rather than time spent within 1 cm of Obj/S1 or S1/S2. Using this alternative analysis, we found multiple intriguing comparisons. For example, cKO mice (p = 0.02), but not WT or cHET mice, spent more time on average in the S2 chamber than the S1 chamber, even though there was no preference for social novelty in any other condition in this study. Furthermore, the effect sizes of analyzing by chamber are smaller than analyzing by time spent within 1 cm of Obj/S1 or S1/S2. Our results imply that chamber dimensions and characteristics are important, but it is unclear what the optimal conditions are for this experiment, even with regards to timing. For example, it is possible that providing more than 10 min with Stranger 1 during the sociability experiment would make Stranger 1 more “familiar” to the experimental mouse, so that when Stranger 2 is introduced during the test for preference for social novelty, there could be a greater contrast between S1 and S2 for the experimental mouse, which might lead to much greater differences in time spent between S1 and S2. This possibility has not been explored in a systematic fashion.

We also found that opposite-sex sociability and preference for social novelty results (Additional file 2: Tables S9, S10) were similar to same-sex sociability and preference for social novelty (Tables 3, 4). A prior study has shown that sociability and preference for social novelty is similar in both females and males, when the stranger mice presented are males [34]. However, to our knowledge, no study to date has shown both same-sex and opposite-sex sociability and preference for social novelty data for both males and females. Our study was not designed to test the nature of opposite-sex sociability or preference for social novelty, so our study cannot provide additional context to our reported results.

At the same time, our study also does not eliminate the possibility that our cHET or even cKO mice are deficient in other, untested behaviors. For example, the full-body *Itgb3* heterozygous knockout shows a higher sensitivity to SSRIs (selective serotonin reuptake inhibitors) in the tail suspension test [28]. That behavior appears to be tied to midbrain serotonergic circuitry however [52], so we would predict that forebrain-specific deletion of *Itgb3* (i.e., in our cHET or cKO mice) would not change sensitivity to SSRIs in the tail suspension test.

## Conclusions

In fine, we demonstrated that deleting *Itgb3* specifically from *Emx1*-expressing cells of the forebrain was sufficient to change self-grooming in novel versus home environments, and to change sociability behaviors in mice. These results are the first to show the behavioral consequences of *Itgb3* loss-of-function in the forebrain, emphasizing its functional importance.

## Methods

### Breeding of mice

To cause *Itgb3* loss of function, *Itgb3*^*fl*/*fl*^ mice [33] on the C57BL6/J background (Jackson Labs #028232) were first crossed with Emx1-Cre^cre/cre^ mice [15] on the C57BL6/J background (Jackson Labs #005628). Then, resulting *Emx1-Cre*^*cre/*+^,*Itgb3*^*fl*/+^ mice (“^+^” refers to the wildtype allele) were crossed with each other to generate conditional knockouts (cKO; *Emx1-Cre*^*cre*^*;Itgb3*^*fl/fl*^), conditional heterozygotes (cHET; *Emx1-Cre*^*cre*^*;Itgb3*^*fl/*+^), and wildtype (WT) controls, consisting of WT Cre- (*Emx1-Cre*^+*/*+^) and WT Cre + (*Emx1-Cre*^*cre*^*;Itgb3*^+*/*+^) on the C57BL6/J background. Thus, two generations of breeding occurred before cKO mice were available. cKO, cHET, and WT mice were all taken from the same generation. Cre-mediated excision of floxed genes in the Emx1-Cre^cre^ mouse occurs prenatally, is robust and efficient [26], and integrin β3 expression in the cerebral cortex of cKO mice is significantly reduced by postnatal day 23 [48], if not earlier. *Emx1* expression occurs in nearly all forebrain excitatory neurons, astrocytes, and oligodendrocytes, particularly in cortex and hippocampus, with sparser *Emx1* expression in the olfactory bulb, amygdala, and piriform cortex [15].

### Housing and testing of mice

WT, cHET, and cKO mice came from six separate litters from the same generation and were 3–6 months old at the time of experiment. Mice were weaned at 3–4 weeks of age with their same-sex littermates into a single standard mouse cage and were not segregated in any other way, including genotype. Two to five weaned mice were kept in each cage, the mean cage occupancy was 3.5 mice, and the median cage occupancy was 3. All mice were always kept on a 12-h light and 12-h dark cycle, and mice were tested during the middle 8 h of the light phase of the cycle. Weaned mice were given Global 18% Protein Rodent Diet (Envigo), access to water at all times, and had 1/4 inch corncob bedding (Envigo). Cage changes occurred once a week throughout the life of the animals. Mice were tested at least 18 h but no more than 5 days after their last cage change. The mean number of days between cage change and testing was 2.9 days, and the median number of days between cage change and testing was 3. Cages were kept on an individually-ventilated cage rack (Allentown). Testing occurred over 11 experimental days (16 calendar days), and 1–5 mice were tested on each experimental day. The sexes and genotypes of the mice tested on each day were randomly determined and experimenters were blind to the genotype of each animal. On the first day that a mouse was tested, it underwent all behavioral tests in the order listed below, starting with the elevated plus maze, with three exceptions: the home cage self-grooming test occurred the next day, and the three-chambered sociability test and three-chambered social novelty test (with male mice as stranger mice) occurred a few weeks later. In other words, mice experienced three experimental days: the first day with the bulk of experiments, the home cage self-grooming test on the very next day, and the sociability and preference for social novelty test (with male mice as the stranger mice) on a day a few weeks later. After completing behavioral experiments, mice were euthanized to collect their brains for further analysis (see Additional file 2: Table S6). In accordance with the James Madison University Institutional Animal Care and Use Committee, and using guidance from the American Veterinary Medical Association Guidelines for the Euthanasia of Animals, mice were fully anesthetized and unconscious following a lethal intraperitoneal injection of ketamine (240 mg/kg)-xylazine (48 mg/kg). Acepromazine (1.85 mg/kg) was also administered with the ketamine-xylazine as a tranquilizer. Once mice were fully anesthetized and unconscious, they were euthanized by transcardial perfusion with ice-cold 1 × phosphate-buffered saline followed by 4% paraformaldehyde in phosphate-buffered saline.

### Sample size calculation

The Mead resource equation [13, 14] was used to estimate a sample size that would be large enough to provide sufficient statistical power for observing an effect in the behavioral tests listed below. This method was chosen over a prospective power analysis because the estimated effect sizes for the planned multifactorial experiments in this study could not be objectively determined. The Mead resource equation aims to maximize the power involving multifactorial animal experimentation by achieving approximately 10–20 error degrees of freedom (DF) in the design of the experiment. Briefly, the error DF is the total DF (in this study, it was the number of mice minus one) minus the model DF (in this study, depending on the experiment, it was five or six). Thus, achieving 20 error DF for all experiments was estimated to require 27 mice, or at least 9 mice per genotype (WT, cHET, and cKO) and at least 14 mice per sex. Six litters were needed to achieve these minimum genotype and sex requirements for a total of 39 mice, and all 39 mice were used for experimentation. Of the 39 mice, 18 were WT (8 female and 10 male), 12 were cHET (8 female and 4 male), and 9 were cKO (5 female and 4 male), for an overall total of 21 female and 18 male mice.

### Overview of behaviors

Adult WT, cHET, and cKO mice underwent experiments that measured repetitive behaviors in home and novel environments (self-grooming), anxiety (elevated plus maze, open field test); hyperactivity and locomotion (open field test), compulsive behaviors (marble burying), and sociability and preference for social novelty (three-chamber social tests). An olfaction test was not conducted because global *Itgb3* knockout mice do not have impaired olfaction [5]. The design of the self-grooming test in a novel environment was replicated after McFarlane et al. [30] and Carter et al. [5]. The design of the elevated plus maze and open field test were adapted from Carter et al. [5]. The design of the marble burying test was adapted from Dohn et al. [11]. The design of the three-chamber social tests was adapted from Moy et al. [34] and Carter et al. [5].

### Order of behavioral experiments

Adult WT, cHET, and cKO mice underwent behavioral testing in the following sequence: elevated plus maze, open field testing, three-chambered sociability test (with a female mouse used as Stranger 1, see below), three chambered social novelty test (with a female mouse used as Stranger 2, see below), novel environment self-grooming test, marble burying test, and home cage self-grooming test. Because experimental mice were of both sexes, all mice were tested in the three-chambered sociability test and three-chambered social novelty test a few weeks later, this time with male mice as Stranger 1 and 2 (S1/S2, see below). The validity of retesting in the three-chambered sociability test has been established by Moy et al. [34]. Similarly, sociability has been shown in opposite-sex (female mice interacting with male S1/S2), and same-sex (male mice interacting with male S1/S2) contexts [34, 35].

### Elevated plus maze

The plus maze consisted of four opaque acrylic arms (each arm 10 cm × 30 cm) connected in a “plus-sign” configuration and elevated approximately 40 cm. Two of the arms had opaque acrylic walls (20 cm H) on three sides and two arms had no walls. The mean illuminance of the maze was approximately 360 lx. A mouse was placed in the center of the maze at the beginning of a 5-min test period with no prior acclimation or exposure to the maze. The position of the mouse was recorded using an overhead video camera. The maze was cleaned with 95% ethanol in between every test run, and once at the beginning of each testing day, to eliminate any odor cues. FIJI software [42] was used to manually analyze the video for the duration of time spent in the open arms, and the percent time spent in the open versus closed arms. When mice in the closed arms approached the open arms, they would sometimes display a “stretched-attend posture” [18] or a “head dip” behavior [49] into the open arms of the maze. Because these behaviors were difficult to distinguish via overhead video, these types of behaviors were combined during analysis and called “peeking”, which was defined as any time the mouse reached into the open arm and returned to the closed arm while maintaining at least one limb in the closed arm section. The duration and the number of peeking behaviors were recorded. Video analysis was done blind to genotype and sex.

### Open field test

The open field test consisted of a mouse being placed in an open-topped, opaque acrylic box (63 cm L × 63 cm W × 63 cm H) that allowed free movement, with no prior acclimation or exposure to the open field. The mean illuminance of the field was approximately 240 lx. The mouse was placed in the center of the box and recorded with an overhead video camera. Mice explored the open field for 15 min, as in Carter et al. [5]. The box was cleaned with 95% ethanol in between every test run, and once at the beginning of each testing day, to eliminate any odor cues. Videos were then manually analyzed using FIJI software to determine the average distance traveled per minute and the number of times the mouse crossed into the middle Sect. (22.5 cm × 22.5 cm) of the field (the middle section was not demarcated physically on the open field, but demarcated during video analysis). Video analysis was done blind to genotype and sex.

### Three chamber sociability test

The three-chamber sociability test involved individual mice being placed in an opaque acrylic box (63 cm L × 63 cm W × 63 cm H) divided into 3 chambers (each 21 cm L × 63 cm W × 63 cm H). Passages (20.7 cm centered along the width of the chamber) allowed for free movement across all chambers. The mean illuminance of the chambers was approximately 240 lx. Chamber 1 (located to the experimenter’s left) contained an inverted wire pencil cup with a female C57BL6/J mouse inside (Stranger 1) that had no prior contact with the experimental mouse. Neither female stranger mice nor female experimental mice were checked for estrus, but experimental mice of all groups were tested over 11 days, so the proportion of experiments with female mice in estrus was assumed to be roughly equal across all groups. The center of the wire cup was placed approximately 10.5 cm away from the leftmost and furthest walls when viewed by the experimenter. The wire cup allowed for nose contact and detection of odor cues but prevented further interaction between mice. Chamber 3 (located to the experimenter’s right) contained an identical inverted wire pencil cup placed approximately 10.5 cm away from the rightmost and furthest walls (when viewed by the experimenter), but without a mouse inside. The location of Stranger 1 was systematically switched between Chambers 1 and 3 between test runs. Each experimental mouse was first acclimated to the three chambers without the presence of pencil cups or Stranger 1 for 10 min. Following acclimation, the experimental mouse was removed, the pencil cups and Stranger 1 were placed in the maze, and then the experimental mouse was placed in the middle chamber and allowed to explore the three chambers for 10 min. The position of the experimental mouse was recorded via overhead video camera. The amount of time spent within 1 cm of each pencil cup and the number of entries into each chamber were recorded by the experimenter, who was blind to genotype. This same test was run on all experimental mice several weeks later, this time using a male C57BL6/J as Stranger 1 (as noted above, the validity of retesting in the three-chambered sociability test has been established by [34]. Videos were manually analyzed for time spent in each chamber and number of trips to each chamber, using FIJI software. Video analysis was done blind to genotype and sex.

### Three chamber social novelty test

Following the sociability test, the experimental mouse was removed from the chamber while another novel C57BL6/J mouse (Stranger 2), unknown to the experimental mouse, was placed under the previously empty pencil cup. The experimental mouse was then placed back into the middle chamber of the three-chamber maze for 10 min of assessment. The experimental mouse could freely explore the chamber and interact with Stranger 1 and Stranger 2. Mice were recorded via overhead video camera. Time spent within 1 cm of each pencil cup and number of entries into each chamber were recorded by the experimenter, who was blind to genotype. As noted above, this same test was run on all experimental mice several weeks later, this time using male C57BL6/J mice as Stranger 1 and 2. Time spent in each chamber and number of trips to each chamber were analyzed later using the video and FIJI software. Video analysis was done blind to genotype and sex.

### Self-grooming in a novel environment

Self-grooming behavior was measured by placing each mouse inside a 37 cm × 23 cm × 22 cm open, empty cage with no bedding. The mean illuminance of the cage was approximately 400 lx. Each mouse was allowed to habituate to the novel environment for 10 min. The time spent self-grooming for the next 10 min was recorded by the experimenter, who was blind to genotype.

### Marble burying test

A novel cage was prepared for each mouse with a 3 cm-thick layer of bedding in order to allow the burying of 1.5 cm diameter marbles. The mean illuminance of the cage was approximately 400 lx. Each mouse was placed in the novel cage without marbles for 10 min of acclimation. Following the acclimation period, each mouse was briefly removed from the cage and 20 marbles were placed in a four-by-five grid on top of the bedding, with 2 cm of space between each marble in all directions. The mouse was then given 10 min to explore and interact with the marbles. After this period the mouse was removed, and marble burying was quantified by the experimenter, who was blind to genotype. Marbles completely buried were given a score of two points while marble partially buried received a score of one point. Marbles that were not buried were given a score of zero points. The marble score was the sum of all the points obtained from the interactions with the 20 marbles.

### Home cage grooming test

After the above behavioral tests were completed, individual mice were placed in a single housed “home cage” and given 24 h to acclimate to it. The cages were kept on a standard cage rack, and ambient light reached the façade of each cage at approximately 200 lx. After a 24-h acclimation period, an observer who was blind to genotype approached the cage without disturbing it in any way and recorded the time the mouse spent self-grooming for 10 min. Afterwards, mice were returned to their original group housing (see "Housing and testing of mice").

### Analysis of data

For self-grooming experiments, the factors involved were environment (home, novel), sex (female, male), genotype (WT, cHET, cKO), and their respective interactions. For sociability and preference for social novelty experiments, the factors involved were chamber (in sociability: Obj, S1; in preference for social novelty: S1, S2), sex (female, male), genotype (WT, cHET, cKO), and their respective interactions. Three-way ANOVA was used to determine if there were any interaction effects among all three factors (e.g., environment, sex, and genotype) or between any factor pairs (e.g., environment and sex, environment and genotype, sex and genotype). Because there were no three-way interactions (see “Results”), two-way ANOVA was used to determine and understand main effects by factor. Repeated measures ANOVA was not used for self-grooming, sociability, or preference for social novelty experiments because there was no significant within-subject interaction in the repeated measure (in all cases, p > 0.5). For EPM, OFT, and MBT, the factors involved were sex and genotype, and two-way ANOVA was used for analysis. When a main effect was found to be p < 0.001 in two-way ANOVA, Šidák's multiple comparisons and within-group ANOVA were used as post-hoc tests. For comparisons between two groups in Additional file 2: Table S5, n = 9 per group was too small to pass the D'Agostino-Pearson normality test, so the data were not assumed to be normally distributed and the Mann–Whitney U-test was used to compare means. SPSS 28 was used to calculate three-way ANOVA and two-way ANOVA. GraphPad Prism 9 was used to confirm two-way ANOVA results in SPSS and for all other statistical testing. GraphPad Prism 9 was used to represent data graphically.

## Supplementary Information

**Additional file 1: Figure S1.** Brains of (A) WT, (B) cHET, and (C) cKO experimental mice following dissection (see Table S6 legend for methods). Scale bar: 0.5 cm.

**Additional file 2: Table S1.** Within-group two-way ANOVA analyses, N, means ± SEM, and three-way ANOVA of self-grooming behaviors. DFn = Degrees of freedom numerator (between-subject degrees of freedom—1); DFd = Degrees of freedom denominator (within-subject degrees of freedom—between-subject degrees of freedom). **Table S2.** Within-group two-way ANOVA analyses, N, and means ± SEM of EPM, OFT, and MBT behaviors. **Table S3.** Within-group two-way ANOVA analyses, N, means ± SEM, and three-way ANOVA of sociability behaviors. **Table S4.** N, means ± SEM and three-way ANOVA of preference for social novelty behaviors. **Table S5.** Within-group N, means ± SEM of behaviors of WT mice expressing Cre recombinase under the control of *Emx1* (Cre +) and WT mice without Cre recombinase expression (Cre−). **Table S6.** Measurements of brain morphology of WT, cHET, and cKO experimental mice following dissection (N, mean ± SEM). Two-way ANOVA (factors: genotype, sex) of each measurement had p > 0.05, except that sex was a significant factor in cortex length (p = 0.0225) and anteromedial-to-posterolateral length in both hemispheres (left p = 0.0397, right p = 0.0231). *Methods*: In accordance with the James Madison University Institutional Animal Care and Use Committee, and using guidance from the American Veterinary Medical Association Guidelines for the Euthanasia of Animals, mice were fully anesthetized and unconscious following a lethal intraperitoneal injection of ketamine (240 mg/kg)-xylazine (48 mg/kg). Acepromazine (1.85 mg/kg) was also administered with the ketamine-xylazine as a tranquilizer. Once mice were fully anesthetized and unconscious, they were euthanized by transcardial perfusion with ice-cold 1 × phosphate-buffered saline followed by 4% paraformaldehyde in phosphate-buffered saline. The brains were then post-fixed in 4% paraformaldehyde in 1 × phosphate-buffered saline until dissected. Measurements of brain morphology were done using a Leica macroscope and FIJI software. Widths were determined by the maximum mediolateral measurement. Lengths were determined by the maximum anteroposterior measurement. “AM-PL Length” refers to the length from the most anteromedial (“AM”) point of the cortex to the most posterolateral (“PL”) point of the cortex; separate measurements were taken for the left hemisphere (“L. Hemi.”) and right hemisphere (“R. Hemi.”). **Table S7.** As in Table S3, except that sociability was measured by time in the *chamber* with the object (Obj) or stranger 1 (S1), rather than time spent near Obj or S1. **Table S8.** As in Table S4, except that preference for social novelty was measured by time in the *chamber* with stranger 1 (S1) or stranger 2 (S2), rather than Obj or S1. **Table S9.** As in Table 3 and Table S3, except that stranger 1 (S1) was the opposite sex of the experimental mouse. **Table S10.** As in Table 4 and Table S4, except that stranger 1 (S1) and stranger 2 (S2) were the opposite sex of the experimental mouse.

## Data Availability

All data are available aggregated with N, mean, and SEM. This permits others to be able to replicate the statistical tests we used and explore the data themselves. Individual data points are shown in the figures; additional individual data points are available from the corresponding author on reasonable request.

## Abbreviations

AMPA: α-Amino-3-hydroxy-5-methyl-4-isoxazolepropionic acid
ANOVA: Analysis of variance
ASD: Autism spectrum disorder
cHET: Conditional heterozygotes for *Itgb3*
cKO: Conditional knockouts for *Itgb3*
DFd: Degrees of freedom denominator (within-subject degrees of freedom—between-subject degrees of freedom)
DFn: Degrees of freedom numerator (between-subject degrees of freedom—1)
EPM: Elevated plus maze
MBT: Marble burying test
NMDA: N-Methyl-d-aspartic acid
Obj: Object (in the three-chambered sociability test)
OFT: Open field test
S1: Stranger mouse #1 (in the three-chambered sociability and preference for social novelty tests)
S2: Stranger mouse #2 (in the three-chambered preference for social novelty test)
SEM: Standard error of the mean
WT: Wildtype
