# Supplementary figures and images for: Integrin β3 in forebrain Emx1-expressing cells regulates repetitive self-grooming and sociability in mice
Source: BMC Neurosci. 2022 Mar 5;23:12. doi: 10.1186/s12868-022-00691-2 (PMC8897866; doi:10.1186/s12868-022-00691-2)

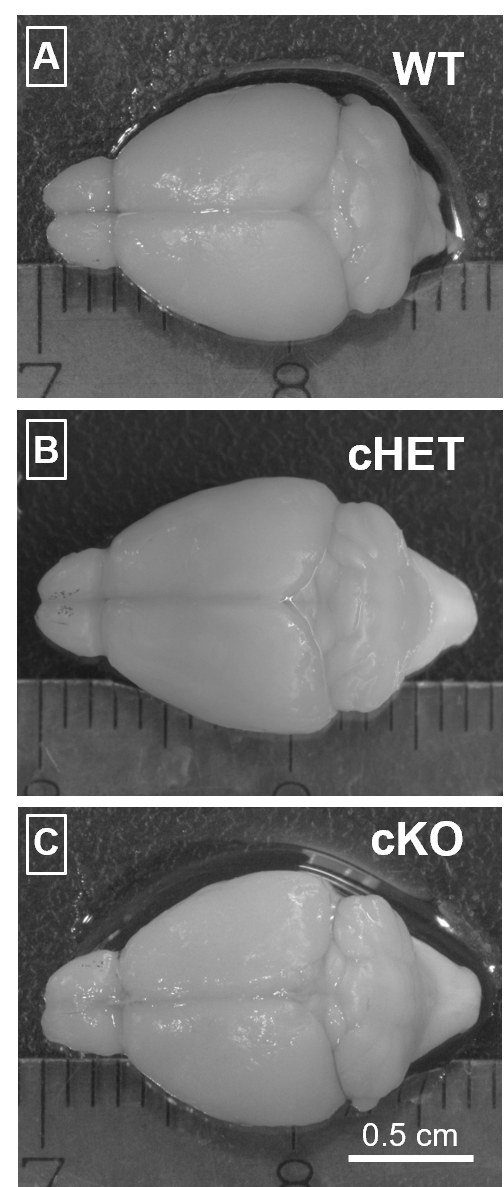

Supplement: Supplementary file 1 — Additional file 1: Figure S1. Brains of (A) WT, (B) cHET, and (C) cKO experimental mice following dissection (see Table S6 legend for methods). Scale bar: 0.5 cm. [file 12868_2022_691_MOESM1_ESM.png]
