# Supplementary material for: Integrin β3 in forebrain Emx1-expressing cells regulates repetitive self-grooming and sociability in mice
Source: BMC Neurosci. 2022 Mar 5;23:12. doi: 10.1186/s12868-022-00691-2 (PMC8897866; doi:10.1186/s12868-022-00691-2)
Supplement: Supplementary file 2 — Additional file 2: Table S1. Within-group two-way ANOVA analyses, N, means ± SEM, and three-way ANOVA of self-grooming behaviors. DFn = Degrees of freedom numerator (between-subject degrees of freedom—1); DFd = Degrees of freedom denominator (within-subject degrees of freedom—between-subject degrees of freedom). Table S2. Within-group two-way ANOVA analyses, N, and means ± SEM of EPM, OFT, and MBT behaviors. Table S3. Within-group two-way ANOVA analyses, N, means ± SEM, and three-way ANOVA of sociability behaviors. Table S4. N, means ± SEM and three-way ANOVA of preference for social novelty behaviors. Table S5. Within-group N, means ± SEM of behaviors of WT mice expressing Cre recombinase under the control of Emx1 (Cre +) and WT mice without Cre recombinase expression (Cre−). Table S6. Measurements of brain morphology of WT, cHET, and cKO experimental mice following dissection (N, mean ± SEM). Two-way ANOVA (factors: genotype, sex) of each measurement had p > 0.05, except that sex was a significant factor in cortex length (p = 0.0225) and anteromedial-to-posterolateral length in both hemispheres (left p = 0.0397, right p = 0.0231). Methods: In accordance with the James Madison University Institutional Animal Care and Use Committee, and using guidance from the American Veterinary Medical Association Guidelines for the Euthanasia of Animals, mice were fully anesthetized and unconscious following a lethal intraperitoneal injection of ketamine (240 mg/kg)-xylazine (48 mg/kg). Acepromazine (1.85 mg/kg) was also administered with the ketamine-xylazine as a tranquilizer. Once mice were fully anesthetized and unconscious, they were euthanized by transcardial perfusion with ice-cold 1 × phosphate-buffered saline followed by 4% paraformaldehyde in phosphate-buffered saline. The brains were then post-fixed in 4% paraformaldehyde in 1 × phosphate-buffered saline until dissected. Measurements of brain morphology were done using a Leica macroscope and FIJI software. Widths were [file 12868_2022_691_MOESM2_ESM.pdf]

Table S1

Two-way ANOVA

within sex

Sex: Female				
Factor	DFn	DFd	F	p
genotype	2	36	0.5997	0.5544
environment	1	36	2.513	0.1216
interaction	2	36	2.409	0.1043

Sex: Male				
Factor	DFn	DFd	F	p
genotype	2	30	1.029	0.3697
**environment**	1	30	**20.31**	**<0.0001**
interaction	2	30	0.5727	0.5701

within genotype

Genotype: WT				
Factor	DFn	DFd	F	p
**environment**	1	32	**16.89**	**0.0003**
sex	1	32	0.8811	0.3549
interaction	1	32	0.02234	0.8821

Genotype: cHET				
Factor	DFn	DFd	F	p
**environment**	1	20	**5.558**	**0.0287**
sex	1	20	0.0867	0.7715
interaction	1	20	0.04854	0.8279

Genotype: cKO				
Factor	DFn	DFd	F	p
environment	1	14	1.464	0.2464
sex	1	14	0.1022	0.754
**interaction**	1	14	**5.901**	**0.0292**

N, mean, SEM

self-grooming time (s)

Genotype: WT			
Condition		n	mean ± SEM
Female	Home	8	35.38 ± 15.48
Female	Novel	8	101.6 ± 23.76
Male	Home	10	23.1 ± 5.896
Male	Novel	10	84.7 ± 15.15

Genotype: cHET			
Condition		n	mean ± SEM
Female	Home	8	56.88 ± 24.32
Female	Novel	8	121.8 ± 23.98
Male	Home	4	41.25 ± 17.45
Male	Novel	4	119.5 ± 49.41

Genotype: cKO			
Condition		n	mean ± SEM
Female	Home	5	83.2 ± 38.24
Female	Novel	5	47.68 ± 17.14
Male	Home	4	21.75 ± 6.408
Male	Novel	4	127.8 ± 37.84

Three-way ANOVA

Factor(s)	F	p
genotype	1.119	0.333
sex	0.116	0.735
environment	16.665	**< 0.001**
genotype, sex	0.257	0.774
genotype, environment	0.535	0.588
sex, environment	3.227	0.077
genotype, sex, environment	2.51	0.089

Table S2

N, mean, SEM

Genotype: WT			
Condition		n	mean ± SEM
EPM Open Entries	Female	8	9.88 ± 2.04
EPM Open Entries	Male	10	8.50 ± 1.26
EPM Percent Time Spent in Open Arms	Female	8	32.95 ± 7.178
EPM Percent Time Spent in Open Arms	Male	10	28.38 ± 4.25
EPM Duration of Peeking Behaviors (s)	Female	8	72.25 ± 10.35
EPM Duration of Peeking Behaviors (s)	Male	10	79.50 ± 6.32
EPM Number of Peeking Behaviors	Female	8	20.75 ± 2.23
EPM Number of Peeking Behaviors	Male	10	20.00 ± 1.317
OFT Distance Traveled (m) per Minute	Female	8	4.33 ± 0.40
OFT Distance Traveled (m) per Minute	Male	10	3.83 ± 0.31
OFT Number of Crossings	Female	8	19.88 ± 2.57
OFT Number of Crossings	Male	10	14.80 ± 2.17
Marble Burying Score	Female	8	10.38 ± 2.154
Marble Burying Score	Male	10	14.40 ± 3.784

Genotype: cHET			
Condition		n	mean ± SEM
EPM Open Entries	Female	8	11.38 ± 1.99
EPM Open Entries	Male	4	8.50 ± 2.99
EPM Percent Time Spent in Open Arms	Female	8	37.26 ± 5.64
EPM Percent Time Spent in Open Arms	Male	4	32.13 ± 10.75
EPM Duration of Peeking Behaviors (s)	Female	8	54.88 ± 6.36
EPM Duration of Peeking Behaviors (s)	Male	4	74.75 ± 21.15
EPM Number of Peeking Behaviors	Female	8	20.38 ± 1.94
EPM Number of Peeking Behaviors	Male	4	21.25 ± 3.64
OFT Distance Traveled (m) per Minute	Female	8	4.96 ± 0.57
OFT Distance Traveled (m) per Minute	Male	4	3.58 ± 0.78
OFT Number of Crossings	Female	8	25.38 ± 6.24
OFT Number of Crossings	Male	4	25.33 ± 6.89
Marble Burying Score	Female	8	9.125 ± 2.622
Marble Burying Score	Male	4	13.0 ± 3.391

Genotype: cKO			
Condition		n	mean ± SEM
EPM Open Entries	Female	8	9.80 ± 2.75
EPM Open Entries	Male	4	8.25 ± 1.931
EPM Percent Time Spent in Open Arms	Female	8	34.70 ± 8.00
EPM Percent Time Spent in Open Arms	Male	4	28.58 ± 3.69
EPM Duration of Peeking Behaviors (s)	Female	8	56.40 ± 12.52
EPM Duration of Peeking Behaviors (s)	Male	4	62.75 ± 10.34
EPM Number of Peeking Behaviors	Female	8	19.20 ± 2.63
EPM Number of Peeking Behaviors	Male	4	18.00 ± 4.02
OFT Distance Traveled (m) per Minute	Female	8	5.02 ± 1.32
OFT Distance Traveled (m) per Minute	Male	4	4.34 ± 0.46
OFT Number of Crossings	Female	8	14.80 ± 6.58
OFT Number of Crossings	Male	4	24.75 ± 6.10
Marble Burying Score	Female	8	8.80 ± 3.262
Marble Burying Score	Male	4	12.25 ± 3.092

Table S3

Two-way ANOVA — within sex

Sex: Female				
Factor	DFn	DFd	F	p
genotype	2	36	2.575	0.0901
chamber	1	36	2.286	0.1393
interaction	2	36	1.075	0.3518

Sex: Male				
Factor	DFn	DFd	F	p
genotype	2	38	1.441	0.2493
**chamber**	1	38	**23.98**	**<0.0001**
interaction	2	38	0.1032	0.9022

Two-way ANOVA — within genotype

Genotype: WT				
Factor	DFn	DFd	F	p
**chamber**	1	34	**13.71**	**0.0008**
**sex**	1	34	**4.811**	**0.0352**
interaction	1	34	0.06829	0.7954

Genotype: cHET				
Factor	DFn	DFd	F	p
**chamber**	1	24	**5.68**	**0.0254**
sex	1	24	0.3839	0.5413
interaction	1	24	0.1398	0.7118

Genotype: cKO				
Factor	DFn	DFd	F	p
chamber	1	16	1.521	0.2353
sex	1	16	0.5081	0.4862
**interaction**	1	16	**4.573**	**0.0483**

N, mean, SEM — time (s) <1 cm of...

Genotype: WT			
Condition		n	mean ± SEM
Female	Obj	8	119.3 ± 28.44
Female	S1	8	179.6 ± 13.77
Male	Obj	11	76.18 ± 8.53
Male	S1	11	145.7 ± 17.33

Genotype: cHET			
Condition		n	mean ± SEM
Female	Obj	8	74.13 ± 25.37
Female	S1	8	130.0 ± 34.98
Male	Obj	6	46.50 ± 11.29
Male	S1	6	123.2 ± 25.06

Genotype: cKO			
Condition		n	mean ± SEM
Female	Obj	5	103.0 ± 13.58
Female	S1	5	87.0 ± 24.13
Male	Obj	5	77.8 ± 15.64
Male	S1	5	137.4 ± 15.45

Three-way ANOVA

Factor(s)	F	p
genotype	3.559	0.033
sex	1.216	0.274
chamber	15.325	**< 0.001**
genotype, sex	1.266	0.288
genotype, chamber	1.084	0.344
sex, chamber	1.823	0.181
genotype, sex, chamber	0.555	0.576

Table S4

N, mean, SEM — time (s) <1 cm of...

Genotype: WT			
Condition		n	mean ± SEM
Female	S1	8	93.50 ± 18.69
Female	S2	8	127.5 ± 26.22
Male	S1	11	106.5 ± 16.36
Male	S2	11	102.5 ± 15.75

Genotype: cHET			
Condition		n	mean ± SEM
Female	S1	8	75.75 ± 22.18
Female	S2	8	85.38 ± 17.91
Male	S1	5	116.6 ± 50.60
Male	S2	5	50.20 ± 13.97

Genotype: cKO			
Condition		n	mean ± SEM
Female	S1	5	43.72 ± 13.17
Female	S2	5	125.7 ± 48.98
Male	S1	5	93.20 ± 23.04
Male	S2	5	101.2 ± 8.59

Three-way ANOVA

Factor(s)	F	p
genotype	1.319	0.274
sex	0.047	0.829
chamber	0.548	0.461
genotype, sex	0.147	0.864
genotype, chamber	1.989	0.144
sex, chamber	4.849	0.031
genotype, sex, chamber	0.225	0.799

Table S5

	WT Cre- (n = 9)	WT Cre+ (n = 9)	
	mean ± SEM	mean ± SEM	p (Mann-Whitney)
Figure 1. Time Self-Grooming (s): Home Environment	38.7 ± 12.7	18.4 ± 7.29	0.185
Figure 1. Time Self-Grooming (s): Novel Environment	108 ± 18.8	76.2 ± 18.1	0.399
Figure 2A. EPM Open Arm Entries	11.22 ± 1.86	7.00 ± 0.87	0.097
Figure 2B. EPM Percent Time in Open Arms	33.66 ± 5.69	27.17 ± 5.38	0.340
Figure 2C. EPM Duration of Peeking Behaviors (s)	73.56 ± 6.77	79.00 ± 9.43	0.504
Figure 2D. EPM Number of Peeking Behaviors	22.22 ± 1.04	18.44 ± 2.02	0.167
Figure 2E. OFT Distance Traveled (m) per Minute	4.570 ± 0.36	3.54 ± 0.26	0.063
Figure 2F. OFT Number of Crossings	19.44 ± 2.48	14.67 ± 2.24	0.118
Figure 2G. Marble Burying Score	15.111 ± 3.96	10.11 ± 2.30	0.308
Figure 3. Time spent within 1 cm of Obj (sociability)	95.56 ± 19.82	93.20 ± 19.31	0.985
Figure 3. Time spent within 1 cm of S1 (sociability)	146.0 ± 17.40	172.6 ± 16.27	0.278

Table S6

N, mean, SEM

Genotype: WT			
Measurement	Sex	n	mean ± SEM
Olfactory Bulb Length (cm)	Female	6	0.2873 ± 0.008959
Olfactory Bulb Length (cm)	Male	10	0.2887 ± 0.007483
Olfactory Bulb Width (cm)	Female	6	0.433 ± 0.002783
Olfactory Bulb Width (cm)	Male	10	0.4294 ± 0.01347
Cerebellum Length (cm)	Female	8	0.3853 ± 0.01337
Cerebellum Length (cm)	Male	10	0.3757 ± 0.02031
Cerebellum Width (cm)	Female	8	0.8846 ± 0.01426
Cerebellum Width (cm)	Male	10	0.8817 ± 0.03438
Cortex Length (cm)	Female	8	0.9523 ± 0.01704
Cortex Length (cm)	Male	10	0.8811 ± 0.02478
Cortex Width (cm)	Female	8	1.022 ± 0.01839
Cortex Width (cm)	Male	10	1.022 ± 0.02668
AM-PL Length, L. Hemi. (cm)	Female	8	1.001 ± 0.006245
AM-PL Length, L. Hemi. (cm)	Male	10	0.9452 ± 0.02763
AM-PL Length, R. Hemi. (cm)	Female	8	0.9915 ± 0.008385
AM-PL Length, R. Hemi. (cm)	Male	10	0.9328 ± 0.02705

Genotype: cHET			
Measurement	Sex	n	mean ± SEM
Olfactory Bulb Length (cm)	Female	8	0.2905 ± 0.008778
Olfactory Bulb Length (cm)	Male	4	0.2492 ± 0.0053
Olfactory Bulb Width (cm)	Female	8	0.4406 ± 0.003164
Olfactory Bulb Width (cm)	Male	4	0.4429 ± 0.009532
Cerebellum Length (cm)	Female	8	0.404 ± 0.01237
Cerebellum Length (cm)	Male	6	0.3876 ± 0.01625
Cerebellum Width (cm)	Female	8	0.8973 ± 0.01249
Cerebellum Width (cm)	Male	6	0.8776 ± 0.02984
Cortex Length (cm)	Female	8	0.9234 ± 0.009499
Cortex Length (cm)	Male	6	0.9229 ± 0.01559
Cortex Width (cm)	Female	8	1.039 ± 0.004597
Cortex Width (cm)	Male	6	1.055 ± 0.01014
AM-PL Length, L. Hemi. (cm)	Female	8	0.9856 ± 0.01179
AM-PL Length, L. Hemi. (cm)	Male	6	0.9866 ± 0.01269
AM-PL Length, R. Hemi. (cm)	Female	8	0.9845 ± 0.008431
AM-PL Length, R. Hemi. (cm)	Male	6	0.9767 ± 0.01127

Genotype: cKO			
Measurement	Sex	n	mean ± SEM
Olfactory Bulb Length (cm)	Female	3	0.2913 ± 0.005609
Olfactory Bulb Length (cm)	Male	5	0.2907 ± 0.01158
Olfactory Bulb Width (cm)	Female	3	0.4294 ± 0.009061
Olfactory Bulb Width (cm)	Male	5	0.4174 ± 0.01393
Cerebellum Length (cm)	Female	3	0.3988 ± 0.009191
Cerebellum Length (cm)	Male	5	0.3955 ± 0.01315
Cerebellum Width (cm)	Female	3	0.9082 ± 0.001365
Cerebellum Width (cm)	Male	5	0.8588 ± 0.0194
Cortex Length (cm)	Female	3	0.9584 ± 0.01478
Cortex Length (cm)	Male	5	0.9057 ± 0.01114
Cortex Width (cm)	Female	3	1.058 ± 0.004557
Cortex Width (cm)	Male	5	1.041 ± 0.009521
AM-PL Length, L. Hemi. (cm)	Female	3	1.018 ± 0.006756
AM-PL Length, L. Hemi. (cm)	Male	5	0.9602 ± 0.01723
AM-PL Length, R. Hemi. (cm)	Female	3	1.013 ± 0.006768
AM-PL Length, R. Hemi. (cm)	Male	5	0.9595 ± 0.01537

Table S7

Two-way ANOVA

time in chamber (s)

Factor	DFn	DFd	F	p
genotype	2	68	0.0007447	0.9993
chamber	1	68	0.8449	0.3613
interaction	2	68	2.701	0.0743

Factor	DFn	DFd	F	p
chamber	1	70	3.134	0.081
sex	1	70	0.3589	0.5511
**interaction**	1	70	**7.324**	**0.0085***

* Šidák's multiple comparisons:			p
Female, Obj	*vs.*	Female, S1	0.9800
Male, Obj	*vs.*	Male, S1	**0.0241**

**Genotype: All**			
Condition		n	mean ± SEM
Female	Obj	21	280.3 ± 40.68
Female	S1	21	249.3 ± 36.57
Male	Obj	16	170.7 ± 13.52
Male	S1	16	319.2 ± 20.23

Factor	DFn	DFd	F	p
genotype	2	68	0.005378	0.9799
sex	1	68	0.2998	0.5858
interaction	2	68	0.02035	0.9799

trips to chamber

Factor	DFn	DFd	F	p
genotype	2	68	0.3517	0.7047
chamber	1	68	0.4912	0.4858
interaction	2	68	0.2457	0.7829

Factor	DFn	DFd	F	p
chamber	1	70	0.6602	0.4192
sex	1	70	0.7198	0.3991
interaction	1	70	0.02445	0.8762

Factor	DFn	DFd	F	p
genotype	2	68	0.8288	0.4409
sex	1	68	1.094	0.2993
interaction	2	68	1.208	0.3052

N, mean, SEM

time in chamber (s)

**Genotype: WT**			
Condition		n	mean ± SEM
Female	Obj	8	204.5 ± 49.86
Female	Mid	8	75.5 ± 16.95
Female	S1	8	322.8 ± 51.62
Male	Obj	8	170.3 ± 22.37
Male	Mid	8	92.88 ± 14.03
Male	S1	8	324.4 ± 30.98

**Genotype: cHET**			
Condition		n	mean ± SEM
Female	Obj	8	326.9 ± 68.68
Female	Mid	8	60.5 ± 16.67
Female	S1	8	207.5 ± 51.03
Male	Obj	4	162.5 ± 32.07
Male	Mid	4	126.5 ± 23.6
Male	S1	4	309.5 ± 52.96

**Genotype: cKO**			
Condition		n	mean ± SEM
Female	Obj	5	327.2 ± 104.3
Female	Mid	5	66 ± 34.49
Female	S1	5	198.6 ± 99.18
Male	Obj	4	179.8 ± 15.48
Male	Mid	4	105.3 ± 19.73
Male	S1	4	318.5 ± 29.13

trips to chamber

**Genotype: WT**			
Condition		n	mean ± SEM
Female	Obj	8	7.625 ± 1.802
Female	Mid	8	15.5 ± 3.257
Female	S1	8	8.875 ± 1.619
Male	Obj	8	7.75 ± 1.623
Male	Mid	8	16.38 ± 2.834
Male	S1	8	8.875 ± 1.302

**Genotype: cHET**			
Condition		n	mean ± SEM
Female	Obj	8	7.25 ± 1.81
Female	Mid	8	15 ± 4.476
Female	S1	8	8.75 ± 2.756
Male	Obj	4	11 ± 4.34
Male	Mid	4	27 ± 12.4
Male	S1	4	16.75 ± 8.148

**Genotype: cKO**			
Condition		n	mean ± SEM
Female	Obj	5	9.2 ± 5.817
Female	Mid	5	16.2 ± 11.85
Female	S1	5	9.6 ± 5.793
Male	Obj	4	9.75 ± 2.056
Male	Mid	4	17.75 ± 3.473
Male	S1	4	8.5 ± 1.19

Table S8

Two-way ANOVA — time in chamber (s)

Factor	DFn	DFd	F	p
genotype	2	60	0.5911	0.5569
**chamber**	1	60	**4.19**	**0.0451***
**interaction**	2	60	**4.113**	**0.0212***

* Šidák's multiple comparisons:			p
WT, S1	*vs.*	WT, S2	0.7088
cHET, S1	*vs.*	cHET, S2	0.5244
cKO, S1	*vs.*	cKO, S2	**0.0200**

**Sex: Both**			
Condition		n	mean ± SEM
WT	S1	15	273.9 ± 28.06
WT	S2	15	232.6 ± 27.82
cHET	S1	9	192.9 ± 26.41
cHET	S2	9	261.2 ± 42.2
cKO	S1	9	191.4 ± 39.58
cKO	S2	9	346.1 ± 52.63

Factor	DFn	DFd	F	p
chamber	1	62	1.219	0.2738
sex	1	62	**0.1412**	**0.7084**
**interaction**	1	62	**4.152**	**0.0459****

** Šidák's multiple comparisons:			p
Female, S1	*vs.*	Female, S2	**0.0373**
Male, S1	*vs.*	Male, S2	0.7891

**Genotype: All**			
Condition		n	mean ± SEM
Female	S1	19	208.3 ± 25.03
Female	S2	19	301.8 ± 31.04
Male	S1	14	257.8 ± 28.61
Male	S2	14	230 ± 32.56

Factor	DFn	DFd	F	p
genotype	2	60	0.5314	0.5905
sex	1	60	0.2068	0.6509
interaction	2	60	0.003724	0.9963

Two-way ANOVA — trips to chamber

Factor	DFn	DFd	F	p
genotype	2	60	0.1499	0.8611
chamber	1	60	0.1324	0.7172
interaction	2	60	0.3089	0.7354

Factor	DFn	DFd	F	p
chamber	1	62	0.04777	0.8277
sex	1	62	3.617	0.0618
interaction	1	62	0.336	0.5642

Factor	DFn	DFd	F	p
genotype	2	60	0.1048	0.9006
sex	1	60	3.137	0.0816
interaction	2	60	0.6079	0.5478

N, mean, SEM — time in chamber (s)

**Genotype: WT**			
Condition		n	mean ± SEM
Female	S1	8	249.4 ± 34.52
Female	Mid	8	82.38 ± 4.5
Female	S2	8	268.9 ± 34.17
Male	S1	7	301.9 ± 45.86
Male	Mid	7	63.86 ± 10.02
Male	S2	7	191.1 ± 42.16

**Genotype: cHET**			
Condition		n	mean ± SEM
Female	S1	6	214 ± 35.49
Female	Mid	6	142.2 ± 60.68
Female	S2	6	252.8 ± 45.96
Male	S1	3	150.7 ± 27.17
Male	Mid	3	174 ± 84.11
Male	S2	3	278 ± 102.9

**Genotype: cKO**			
Condition		n	mean ± SEM
Female	S1	5	135.8 ± 59.07
Female	Mid	5	44.6 ± 17.61
Female	S2	5	413.4 ± 75.94
Male	S1	4	261 ± 26.07
Male	Mid	4	136.8 ± 29.23
Male	S2	4	262 ± 52.59

N, mean, SEM — to chamber

**Genotype: WT**			
Condition		n	mean ± SEM
Female	S1	8	9.625 ± 0.5957
Female	Mid	8	18.13 ± 1.747
Female	S2	8	9.5 ± 1.323

**Genotype: cHET**			
Condition		n	mean ± SEM
Female	S1	6	11.17 ± 4.84
Female	Mid	6	13 ± 3.435
Female	S2	6	8.167 ± 2.496

**Genotype: cKO**			
Condition		n	mean ± SEM
Female	S1	5	7.6 ± 4.226
Female	Mid	5	14.2 ± 7.365
Female	S2	5	7.2 ± 3.323

	trips	Male	S1	7	6.286 ± 1.286
		Male	Mid	7	12.14 ± 2.613
		Male	S2	7	6.571 ± 1.478

Male	S1	3	5.667 ± 2.728
Male	Mid	3	10 ± 4.359
Male	S2	3	5 ± 1.732

Male	S1	4	6.25 ± 0.8539
Male	Mid	4	14.5 ± 3.069
Male	S2	4	8 ± 1.581

Table S9

Two-way ANOVA

Factor	DFn	DFd	F	p
genotype	2	84	0.859	0.4273
**chamber**	1	84	**18.92**	**<0.0001***
interaction	2	84	0.3583	0.6999

* Šidák's multiple comparisons:			p
WT, Object	*vs.*	WT, Stranger 1	**0.0052**
cHET, Object	*vs.*	cHET, Stranger 1	**0.0151**
cKO, Object	*vs.*	cKO, Stranger 1	0.2894

Factor	DFn	DFd	F	p
**chamber**	1	86	**21.58**	**<0.0001****
sex	1	86	2.447	0.1214
interaction	1	86	0.9864	0.3234

** Šidák's multiple comparisons:			p
Female, Object	*vs.*	Female, Stranger 1	**0.0211**
Male, Object	*vs.*	Male, Stranger 1	**0.0003**

Factor	DFn	DFd	F	p
genotype	2	84	0.6836	0.5076
sex	1	84	2.182	0.1434
interaction	2	84	0.2715	0.7629

within sex

**Sex: Female**				
Factor	DFn	DFd	F	p
genotype	2	40	0.8836	0.4212
**chamber**	1	40	**9.551**	**0.0036**
interaction	2	40	0.5517	0.5803

**Sex: Male**				
Factor	DFn	DFd	F	p
genotype	2	38	0.3988	0.6739
**chamber**	1	38	**8.076**	**0.0072**
interaction	2	38	0.4963	0.6126

within genotype

**Genotype: WT**				
Factor	DFn	DFd	F	p
**chamber**	1	34	**13.61**	**0.0008**
sex	1	34	0.2149	0.6459
interaction	1	34	3.61	0.0659

**Genotype: cHET**				
Factor	DFn	DFd	F	p
chamber	1	24	**7.496**	**0.0115**
sex	1	24	1.161	0.2919
interaction	1	24	0.0004377	0.9835

**Genotype: cKO**				
Factor	DFn	DFd	F	p
chamber	1	20	1.614	0.2185
sex	1	20	0.994	0.3307
interaction	1	20	0.01395	0.9072

within chamber

**Chamber: Object**				
Factor	DFn	DFd	F	p
genotype	2	39	1.439	0.2496
sex	1	39	0.6105	0.4393
interaction	2	39	1.404	0.2577

**Chamber: Stranger 1**				
Factor	DFn	DFd	F	p
genotype	2	39	0.07517	0.9277
sex	1	39	2.087	0.1565
interaction	2	39	0.03284	0.9677

N, mean, SEM

time (s) <1 cm of...

**Genotype: WT**			
Condition		n	mean ± SEM
Female	Obj	8	115.8 ± 21.98
Female	S1	8	152.3 ± 14.19
Male	Obj	11	86.45 ± 15.16
Male	S1	11	200.5 ± 24.75

**Genotype: cHET**			
Condition		n	mean ± SEM
Female	Obj	8	64.38 ± 18.65
Female	S1	8	148.3 ± 35.76
Male	Obj	6	97 ± 25.36
Male	S1	6	182.2 ± 39.36

**Genotype: cKO**			
Condition		n	mean ± SEM
Female	Obj	7	103.3 ± 11.79
Female	S1	7	158.7 ± 26.5
Male	Obj	5	147.8 ± 61.24
Male	S1	5	193.8 ± 61.48

Table S10

Two-way ANOVA

Factor	DFn	DFd	F	p
genotype	2	84	0.249	0.7802
chamber	1	84	0.339	0.5293
interaction	2	84	1.023	0.364

Factor	DFn	DFd	F	p
chamber	1	86	0.3193	0.5735
sex	1	86	2.986	0.0876
interaction	1	86	0.4594	0.4497

Factor	DFn	DFd	F	p
genotype	2	84	0.1067	0.8989
sex	1	84	3.004	0.0867
interaction	2	84	0.8154	0.4459

N, mean, SEM

time (s) <1 cm of...

Genotype: WT			
Condition		n	mean ± SEM
Female	S1	8	111.4 ± 22.81
Female	S2	8	119.9 ± 21.45
Male	S1	11	119.5 ± 19.15
Male	S2	11	123.9 ± 15.96

Genotype: cHET			
Condition		n	mean ± SEM
Female	S1	8	105.5 ± 25.59
Female	S2	8	76.25 ± 19.8
Male	S1	6	130.3 ± 13.56
Male	S2	6	136 ± 31.81

Genotype: cKO			
Condition		n	mean ± SEM
Female	S1	7	98.29 ± 13.12
Female	S2	7	117.6 ± 11.3
Male	S1	5	99.6 ± 14.83
Male	S2	5	148.4 ± 36.76
